# A gastrointestinal stromal tumor with mesenteric and retroperitoneal invasion

**DOI:** 10.1186/1477-7819-5-121

**Published:** 2007-10-24

**Authors:** Gulgun Engin, Oktar Asoglu, Yersu Kapran, Gulsen Mert

**Affiliations:** 1 Department of Radiology, Istanbul University, Istanbul Faculty of Medicine, Istanbul, Turkey; 2 Department of General Surgery, Istanbul University, Istanbul Faculty of Medicine, Istanbul, Turkey; 3 Department of Pathology, Istanbul University, Istanbul Faculty of Medicine, Istanbul, Turkey

## Abstract

**Background:**

Gastrointestinal stromal tumors are rare visceral sarcomas arising in the gastrointestinal tract wall. In this report we present a case of gastrointestinal stromal tumors with mesenteric and retroperitoneal invasion, describe and discuss its computed tomography findings.

**Case presentation:**

A 57-years-old male patient has been complaining of abdominal distention, weight lose, and hematuria. During physical examination, significant distention and multiple palpable tumor masses were identified on the abdomen. Abdominal computed tomography showed multiple, well-defined, soft tissue masses with homogenous and heterogeneous pattern, in the mesenteric and retroperitoneal areas. Unlike specific features of gastrointestinal stromal tumor, renal obstruction and atypical central calcification without chemotherapy that has not been yet described were seen in this case. Computed tomography did not reveal liver metastases and/or the lymph nodes with pathological size. Ultrasonography-guided true-cut^® ^biopsy was made, histopathologic and immunohistochemical analyses demonstrated stromal tumor which, C-KIT (+). The patient underwent left ureterectomy, left nephrectomy and total colectomy. Postoperative histopathological analyses revealed lower grade malignant GISTs. As of 17 months after the surgery, he is alive and free of recurrence.

**Conclusion:**

When intraabdominal, multiple, large (>5 cm), well-circumscribed, homogenous or heterogeneous mass lesions without ascites, omental caking and lymph nodes metasteses were seen, gastrointestinal stromal tumors should be considered in the differential diagnosis.

## Background

Gastrointestinal (GI) stromal tumor (GIST) is a rare visceral sarcoma arising in the gastrointestinal tract wall [1,2]. This tumor arises in the muscularis mucosa and muscularis propria layers anywhere from the esophagus to the rectum. Its most common anatomic sites of origin are the stomach (60–70%), small intestine (20–30%), colon and rectum (5%), abdominal cavity, i.e. peritoneum and omentum (5%), esophagus (<5%) and retroperitoenal space (<3%) [3-5] In this report we present a rare case of multiple primary mesenteric and retroperitoneal GISTs, describe and discuss its computed tomography (CT) findings.

## Case presentation

**Figure 1:**
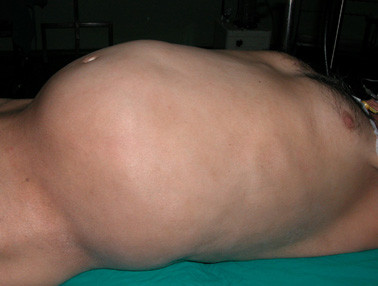
Preoperative appearance of the patient showing abdominal distention.

**Figure 2:**
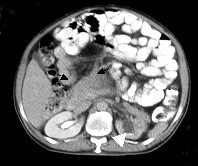
Abdominal CT image shows homogenous, well-defined, mass lesion in the retroepritoneal area (black arrows). Grade 3 hydronephrosis in the left kidney is shown with white arrowhead.

**Figure 3:**
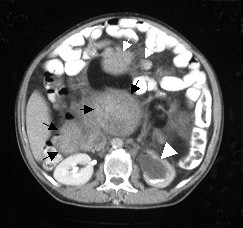
Abdominal CT image of slightly more distal of figure 2 shows multiple, mesenteric (white small arrows) and retroperitoneal (black arrows), well-defined, homogenous, solid masses. There is also grade 3 hydronephrosis in the left kidney (white arrowhead)

**Figure 4:**
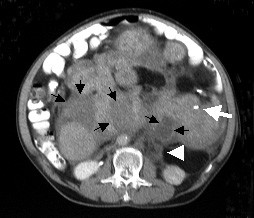
Abdominal CT image of slightly more distal of figure 3 shows multiple, homogenous or central necrotic (black arrows) or calcific (white arrow) masses invaded to the small bowel mesentery and left ureter (arrowhead).

A 57-years-old male patient was admitted to the general surgery department with abdominal distention, weight loss and hematuria. During physical examination, significant distention and multiple palpable tumor masses were identified on the abdomen (Figure 1). Contrast-enhanced abdominal multi detector CT imaging was performed with a four-detector row CT scanner (Somatom Sensation 4^®^, Siemens Medical Solutions, Erlangen, Germany). Multiple, well-defined, soft tissue masses with homogenous pattern, measuring maximum 6 × 5 cm in size were observed in the mesenteric and retroperitoneal areas (Figure 2, 3). In some masses, central necrosis and coarse calcifications were seen (Figure 4). There was grade 3 hydronephrosis in the left kidney due to the left ureteric invasion (Figure 2, 3, &4). However, CT did not reveal liver metastasis and/or the lymph nodes with pathological size.

**Figure 5:**
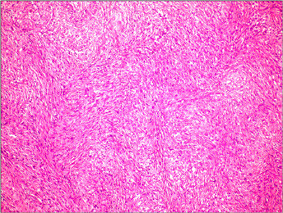
In microscopic appearance of histopathologic analyses, GIST has spindle cell phenotype in a storiform array. Note the compact and highly   cellular pattern, mild-diffuse atypia (H&E, W100).

**Figure 6:**
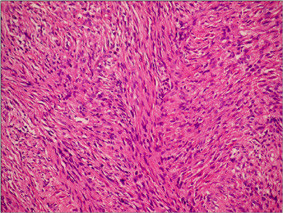
In microscopic appearance of histopathologic analyses, GIST cells   have paler eosinophilic cytoplasm with indistinct cell margins and minimal   tumor stroma  (H&E, W200).

**Figure 7:**
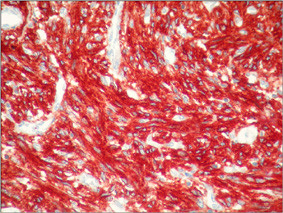
In microscopic appearance of immunohistochemical analyses, CD117   immunoreactivity is shown as diffuse strong cytoplasmic staining in spindle   cell (CD117 immunostain, W200).

**Figure 8:**
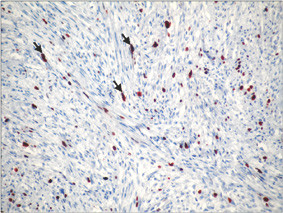
Microscopic appearances of immunohistochemical analyses. Approximately 15% of tumor cells show nuclear positivity for Ki-67 (MIB-1) (arrows) (Ki-67 immunostain, ×200).

**Figure 9:**
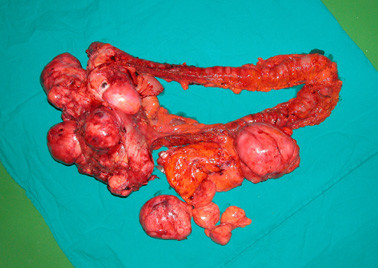
Multiple tumoral nodules in colon serosa and mesocolon are shown in gross specimen after performing total colectomy.

**Figure 10:**
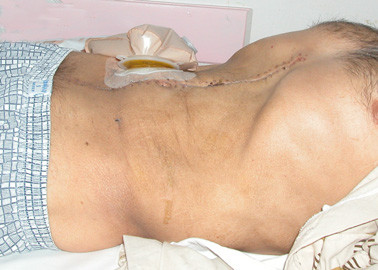
Postoperative appearance of the patient. Abdominal distension has been significantly reduced after the masses have been totally removed. Incision scar and an end ileostomy bag are seen.

Ultrasonography (US)-guided true-cut^® ^biopsy was made, histopathologic (Figure 5, 6) and immunohistochemical analyses demonstrated stromal tumor which, C-KIT (+)(Figure 7), CD34 (-), S-100 (-), desmin (-), actine (-) Ki-67 (+)(Figure 8) and with a proliferation index of <%1. During the operation, not only the tumor filling the whole abdomen but masses invading small bowel mesentery, left hemicolon and the left ureter have been detected. The patient underwent left ureterectomy, left nephrectomy and total colectomy. Proximally from the treitz, small bowel resection from a distance of 200 cm was applied. The mass have been totally removed and taken out of the abdomen (Figure 9). The operation has been completed with an end-ileostomy (Figure 10).

Postoperative histopathological analyses revealed that the tumor was total 25 cm in diameter. It was composed of spindle-shaped cells with hemorrhagic areas. Mitotic activity was 3 mitoses per 50 HPF. The tumor invaded the small bowel serosa, colonic submucosa and the left ureter. Thus the tumor was not described as an extra-GIST that arose from the soft tissue of the abdominal cavity (omentum and mesentery) and retroperitoneum because of the existance of an attachment of serosa of GI tract [4]. The tumor was described as a GIST with predominant mesenteric and retroperitoneal invasion and classified as a lower grade GIST according to Bucher grading system for GIST after surgical resection [6].

He had an uneventful postoperative course and was discharged home on postoperative day 3. As of 17 months after the surgery, he is alive and free of recurrence.

## Discussion

CT, magnetic resonance imaging (MRI) and fluorine-18-fluorodeoxyglucose (FDG) positron emission tomography (PET) can be used for evaluation of GIST. Contrast-enhanced CT scan is, however, more widely available and is currently the imaging modality of choice for patients with suspected abdominal mass or biopsy-proven GIST. CT is used in staging and surgical planning. When a small tumor is found incidentally during endoscopy, the local extent of the tumor should be evaluated using either endoscopic ultrasound or CT. For those with known or suspected rectal GIST, a dedicated MRI provides better information than CT scan in preoperative staging work-up. Evaluation of FDG uptake using PET scanning is recommended when an early detection of tumor response to imatinib treatment is required [7].

CT findings of GISTs are well described in the radiological literature [8-13]. Malignant GISTs are typically extraluminal, large (>5 cm), well-circumscribed, heterogeneous, centrally necrotic tumors. Such features contrast with the regular contours, homogeneous density, and intraluminal growth patterns that are characteristic of smaller benign GISTs. Thus, well-circumscribed tumors appear to be a feature of GISTs, as in our case, on CT imaging. Heterogeneous density, on the other hand, was independent from tumor dimension. Heterogeneous density, pathologically concordant with hemorrhage, has been found in tumors less than 5 cm, despite the homogeneous densities found in the tumors bigger than 5 cm.

Exceptional to the reports published so far, Takao *et al*., reported a GIST of the peripancreatic retroperitoneum with CT and MR imaging findings representing huge well-defined inhomogeneous mass with peripheral calcification [14]. In our case, atypical central course calcification without chemotherapy that has not been yet described, were seen in only one mass.

The spread pattern of these tumors shows predilection for liver metastasis and peritoneal dissemination [14]. In that case, however, concordant with the results of a few studies published previously, the relationship between multiple tumors and metastasis was not seen. No liver or lymph node metastasies have been seen in spite of multiple malign stromal tumor masses [5,11].

Burkill *et al*., reported that stromal tumors rarely obstruct viscera, despite their large size [10]. In our case, no small bowel or colonic obstruction findings have been found in spite of the proximity of mesenteric multiple mass lesions to the colon, – especially descending colon-. However, in that case, there is a hydronephrosis due to ureteral obstruction which is a very rare finding. In the report of Burkill *et al*., 116 malignant GIST were analyzed, and only one case has this behavior [10].

In our case, we have seen multiple peritoneal tumor masses without peritoneal fluid. Burkill, *et al*., concordantly with our case, reported that ascites and omental caking were unusual findings in stromal tumors [10].

Tumor size, anatomic location, and mitotic count are considered independent prognostic factors for GISTs [15,16], and tumors that measure ≥5 cm are associated with an unfavorable prognosis. The anatomic site of origin of a GIST is associated strongly with its clinical behavior, and patients who have tumors that arise in the rectum or small intestine have the worst prognosis compared with the more favorable outcomes observed among patients who have esophageal and gastric neoplasms [16]. Likewise, GISTs that exhibit ≥5 mitoses per 50 HPF or ≥2 mitotic figures per 10 HPF are associated with an unfavorable prognosis regardless of their site of origin [15,17-22]. Our case had multiple mesenteric and retroperitoneal stromal tumors; mitoses were < 5 per 50 HDI, total tumor diameter was 25 cm. The localization was unfavorable but bad prognostic signs such as liver or lymph node metastases were not seen.

The differential diagnosis of an abdominal mass with well defined, extraluminal masses and homogeneous enhancement in the abdominal cavity includes peritoneal implants, lymphoma, primary peritoneal carcinomatosis, carcinoids, and other soft-tissue tumors (leiomyosarcoma, malignant tumors of nerve sheath and vascular origin) [10,12,23]. In our case, absence of peritoneal ascites and omental caking excluded peritoneal implant and primary peritoneal carcinomatosis, absence of lymph node metastases excluded lymphoma diagnosis. For definitive diagnosis, US-guided true-cut^® ^biopsy and then histopathologic and immunohistochemical analyses were made. However, preoperative percutaneous biopsy carries a theoretical risk of peritoneal seeding or tumor rupture, and is indicated only for clearly irresectable disease or when treatment needs to be altered, as would be the case if the mass proved to be lymphoma or germ cell tumor [24]. One alternative technique is endoscopic US-fine needle aspiration (FNA), which has been used increasingly for the evaluation of different types of intraabdominal and intrathoracic lesions. Observations to date indicate that endoscopic US-FNA is a safe and accurate procedure [25].

Immunoreactivity for CD117 (C-KIT) is a characteristic of GISTs; in addition, most GISTs express CD34 immunostain. Another marker that is considered useful in the assessment of GISTs is Ki-67 immunostain, with reported 100% specificity and sensitivity for malignancy on GISTs [26]. Immunohistochemical studies on that tumor cells revealed positive staining for C-KIT (CD117), whereas CD34, antibodies against smooth-muscle actin (SMA), desmin, actine and S-100 protein were negative.

## Conclusion

When intraabdominal, multiple, large (>5 cm), well-circumscribed, homogenous or heterogeneous mass lesions without ascites, omental caking and lymph nodes metasteses were seen, GIST should be considered in the differential diagnosis.

## Competing interests

The author(s) declare that they have no competing interests.

## Authors' contributions

CT examinations and writing of this case report were performed by Gulgun Engin. The case was operated by Oktay Asoglu and histopathological and immunohistochemical analyses were performed by Yersu Kapran. Gulsen Mert was contributed to the study by reviewing the pathology of tumor and by taking photos of the microscopic appearances of the tumor.
